# A retrospective analysis for the management of oromaxillofacial invasive mucormycosis and systematic literature review

**DOI:** 10.1186/s12903-023-02823-4

**Published:** 2023-02-21

**Authors:** Chen-xi Li, Zhong-cheng Gong, Parekejiang Pataer, Bo Shao, Chang Fang

**Affiliations:** 1 Department of Oral and Maxillofacial Oncology and Surgery, The First Affiliated Hospital of Xinjiang Medical University, School/Hospital of Stomatology, Xinjiang Medical University, Stomatological Research Institute of Xinjiang Uygur Autonomous Region, No.137 Liyushan South Road, Ürümqi, 830054 People’s Republic of China; 2 Hubei Province Key Laboratory of Oral and Maxillofacial Development and Regeneration, Wuhan, 430022 People’s Republic of China

**Keywords:** Mucormycosis, Therapeutic regimen, Maxillofacial area, Systematic review

## Abstract

**Purpose:**

Mucormycosis is a type of fatal infectious disease, rarely involved in the oromaxillofacial region. This study aimed to describe a series of 7 cases with oromaxillofacial mucormycosis and to discuss the epidemiology, clinical features, and treatment algorithm thereof.

**Methodology:**

Seven patients in the author’s affiliation have been treated. They were assessed and presented as per their diagnostic criteria, surgical approach, and mortality rates. Reported cases of mucormycosis originally happened in craniomaxillofacial region were synthesized through a systematic review so as to better discuss its pathogenesis, epidemiology, and management.

**Results:**

Six patients had a primary metabolic disorder, and one immunocompromised patient had a history of aplastic anemia. The criteria for a positive diagnosis of invasive mucormycosis were based on clinical presentation of signs and symptoms, and a biopsy for microbiological culture and histopathologic analysis. Each patient used antifungal drugs and five of them also underwent surgical resection at the same time. Four patients died due to the unregulated spread of mucormycosis, and one patient died owing to her main disease.

**Conclusions:**

Although uncommon in clinical practice setting, mucormycosis should be of great concern in oral and maxillofacial surgery, due to the life-threatening possibility of this disease. The knowledge of early diagnosis and prompt treatment is of utmost importance for saving lives.

**Supplementary Information:**

The online version contains supplementary material available at 10.1186/s12903-023-02823-4.

## Introduction

Mucormycosis is a saprophytic opportunistic, but a highly aggressive fungal infection caused by the class Zygomycetes, belonging to members of Mucorales order, subphylum *Mucoromycotina* [1]. Mainly, the genera of *Absidia*, *Mucor*, *Rhizomucor*, and *Rhizopus* are responsible for its aetiology, although *Apophysomyces*, *Cunninghamella*, and *Saksenaea* can also be associated pathogenic species [2, 3]. It is an uncommon, rapidly emerging infection of fungi, with high morbidity and mortality that can produce widespread oral-maxillofacial tissue necrosis [4, 5]. Due to the rarity of this disease (especially occurred in craniofacial units), it is almost impossible to conduct multicentric and randomized clinical trials with large sample size; hence, most of the existent data in respect of epidemiological characteristics, diagnostic strategy, and therapeutic decision-making originate from case series and case reports.

According to the published literature, the prevalence of mucormycosis is the highest in India that stands approximately at 14 per 100,000 populations, whereas it varies between 0.01 and 0.2 for each 100,000 population in European countries and the United States [2]. However, the information on the incidence of oromaxillofacial mucormycosis manifestations is limited. Reported cases presented mucoraceous lesions in the rhino-orbito-cerebral (34%), pulmonary (20%), cutaneous (22%), disseminated (13%), gastrointestinal (8%), and other unusual rare sites (3%) including renal, middle ear, parotid gland, mediastinum, heart and valves, uterus, urinary bladder, cervical lymph nodes, and even oral cavity [6, 7]. Following the initial infection, mucormycosis typically progresses quickly, with rapid invasion of blood vessels resulting in tissue necrosis and thrombosis [8, 9].

The diagnosis and treatment of oromaxillofacial mucormycosis is difficult because of its scarcity. Grasping the magnitude of this formidable challenge would help advance our understanding of oromaxillofacial mucormycosis. Our study, therefore, sought to investigate the diagnostic criteria and treatment approach in the Department of Oromaxillofacial Oncology and Surgery, the First Affiliated Hospital of Xinjiang Medical University, Urumqi, China between 2013 and 2021, through 7 rare cases that demonstrate oromaxillofacial mucormycosis; as well as update and collate the review of literature regarding mucormycosis, exploring the epidemiological characteristics, course of infection, treatment regimens, and clinical outcomes.

The present study was approved by the Ethics Committee of the First Affiliated Hospital of Xinjiang Medical University (approval No. 52966-05/08/2022).

## Methods

On the whole, 7 cases admitted to our department from 2013 to 2021 were enrolled in this study. For the objective of guiding internal medication, all patients were assigned to consult a clinical pharmacist group as well. Clinical presentation of signs and symptoms, blood routine examination, blood biochemistry indexes, liver/kidney function tests, radiographic imaging, treatment procedure, post-operative histopathological findings, pathogenic microbes’ culture and antimicrobial susceptibility test, and survival and prognosis were retrospectively analyzed and reported. The study protocol was approved by the Ethics Committee, the First Affiliated Hospital of Xinjiang Medical University and followed the principles outlined in the Declaration of Helsinki. Written informed consent to participate in this study was provided by the participants or legal guardian/next of kin. All data generated or analyzed during this study are included in this published article.

## Results

**Fig. 1:**
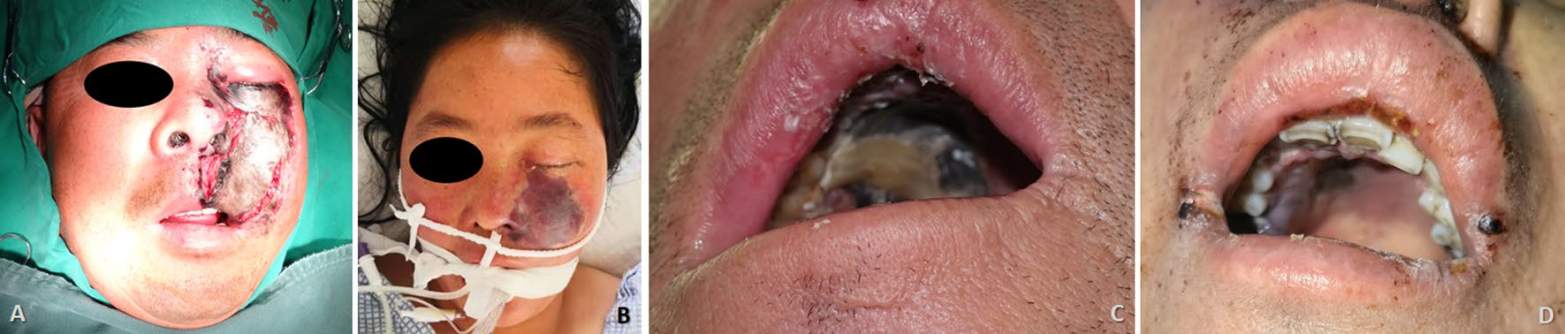
Extraoral appearance of invasive mucormycosis seen in **A** and **B**; intraoral appearance of invasive mucormycosis seen in **C** and **D**

**Fig. 2:**
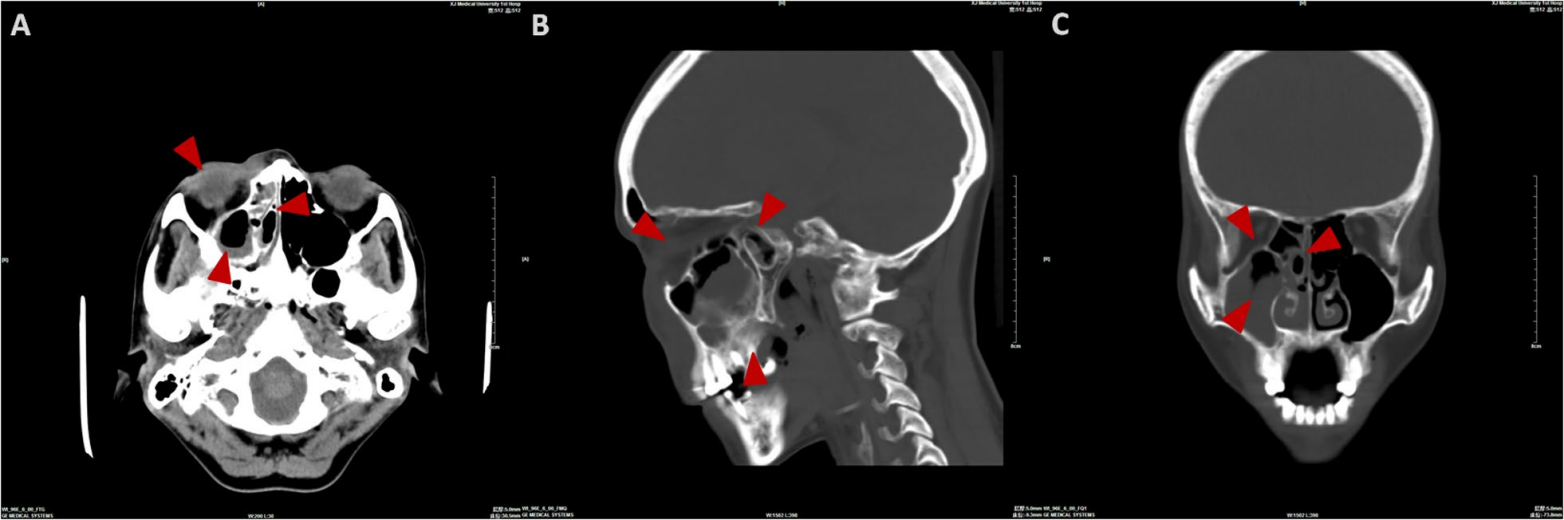
Computerized tomography scanning (**A** axial image; **B** sagittal image; **C** coronal image) showed swelling of soft tissue in right maxillofacial region, heterogeneous opacification of subcutaneous fat space, in-sinus low density in ethmoid, frontal, sphenoid, maxillary with partial destruction of sinuses’ wall. Scope of infection was indicated by the arrows

In this case series, female patients were affected less frequently than the males with a ratio of 2:5. The median age of patients was 45.0 year-old, ranging from 27 to 63. All seven patients were moderately built and moderately nourished, of which six were known as a history of poorly controlled diabetes mellitus without regular treatment (insulin injection/oral hypoglycemic medication); and one patient was diagnosed with severe aplastic anemia (Table 1). Three of these patients had undergone tooth extraction under local anesthesia but without any complications. The lesion of all patients were characterized by extensive ulceration involved orbital part, nasal cavity, maxilla, maxillary sinus, hard palate and alveolar process, resulting in exposed bone and teeth exfoliation (Fig. 1). Possible orbital cellulitis, osteomyelitis as well as maxillary sinusitis mimicking pain or toothache presented in all 7 cases (100%) and fever in 3 cases (42.86%) (Table 1). Total features of craniomaxillofacial computerized tomography (CT) scanning our patients revealed clouding haziness in the maxilla; maxillary sinus thickening/fullness; extensive soft tissue swelling (Fig. 2).

**Table 1:** Clinical information of the patients

Order (time)	Sex	Race	Age (years)	Primary disease	Exodontics history	Presentation of signs and symptoms	Blood routine examination	Serum biochemical test	CT(A)/MRI results	Treatment	Prognosis
1(2013)	Male	Han nationality	46	Uncontrolled type-2 diabetes mellitus	No	Blackish necrotic skin with stink covering infraorbital region; fever; blurred vision in right eye; severe pain and swelling in the left posterior maxilla with proptosis of the left globe and ophthalmoplegia (restricted motion of eyeball); the gum in the left maxilla had severe tenderness	WBC-12.74; NEUT%-87.10; neutrophils-21.82; Hct-37.0%; PLT-93	IL 6-93.190; PCT-0.37; BUN-32.0; ESR-40.0; HbA1c-12.3%; Scr-2.28; FBG-12.68; PPBS-64	Diffuse mucosal thickening in both maxillary sinuses and frontal sinuses, cavernous sinus thrombosis, embolization of the maxillary artery, necrosis of the left maxilla and infraorbital area, subarachnoid hemorrhage	Surgical debridement; Antimycotic agent (NA)	Died
2(2018)	Male	Han nationality	27	Hyperglycemia	Maxillary anterior left incisor	Left pupil Dilation; blackish ala nasi; a mild diffuse swelling over the left middle third of the face which was extending mediolaterally from the lateral aspect of nose to the outer canthus of the eye and superoinferiorly from the infraorbital region to 1 cm above the corner of the mouth	WBC-25.05; NEUT%-83.90; neutrophils-9.99; Hct-38.3%; PLT-474	IL 6-295.600; PCT-0.52; CRP-162.74; BUN-5.43; Scr-82.11	Flow void through the left ophthalmic artery and superior ophthalmic vein, absence of enhancement within the left medial and lateral rectus muscles and along the optic nerve/sheath, left maxillary sinus and nasal mucosa thickening	Surgical debridement, removal of left nostril inferior concha, partial ostectomy of maxillary sinus; Compound fungicides (NA)	Alive
3(2020)	Male	Kazakh	52	Uncontrolled type-2 diabetes mellitus	Multiple maxillary teeth	Right orbital sanguineous discharge; medial canthus malposition with necrosis extending to the ipsilateral ala nasi; dull aching pain with intermittent extra oral swelling over right maxilla and numbness of right side of upper lip	WBC-16.82; NEUT%-86.00; neutrophils-14.47; Hct-29.2%; PLT-475	IL 6-196.300; PCT-0.28; CRP- > 90.00; DD-426.0; BUN-2.75; FBG-4.95; PPBS-133.66; Scr-35.18	Right globe proptosis with asymmetric retrobulbar fat stranding and extensive opacification of right maxillary, ethmoid, and frontal sinuses; partial opacification of the right sphenoid sinus and erosions of the lamina papyracea	Surgical debridement with partial maxillectomy; Intravenous liposomal AmB	Recurrence died
4(2020)*	Female	Uygur	63	Metabolic acidosis (diabetic ketoacidosis)	Maxillary right 1st molar, multiple extractions in mandible	Right zygomaticofacial defect by necrosis; exposed necrotic gray-colored bone in the maxillary molar region, with surrounding necrotic tissue	WBC-16.54; NEUT%-91.70; neutrophils-7.65; Hct-33.4%; PLT-119	IL 6-27.420; PCT-0.21; CRP- > 90.00; BUN-5.95; FBG-15.69; PPBS-51.00; Scr-92.55	The patient continued to deteriorate, was ventilated, and eventually required inotropic support. Due to persistent hypotension, we were unable to carry out repeat imaging or any debridement measures. Despite all measures, she died unfortunately on day six of this admission		
5(2021)	Male	Uygur	43	Uncontrolled type-2 diabetes mellitus	No	Grey-black-colored ala nasi; pain in the upper right posterior tooth region; edema and tenderness of the left cheek; left maxillary swelling, vestibular swelling, pain and necrosis of gum; fever	WBC-16.85; NEUT%-83.90; neutrophils-13.88; Hct-42.5%; PLT-347	IL 6-176.600;CRP-59.6; BUN-5.30; FBG-15.21; Scr-78.30	Opacification of the left maxillary sinus, ethmoid sinus, and frontal sinus, early stage cavernous sinus thrombosis, erosion of the anterolateral wall of left maxillary antrum with thickening of the sinus lining with moth eaten appearance of the maxillary bone seen, left facial artery and ophthalmic artery were not developed indicating embolisms existence	Specific antifungal therapy was performed****; Surgical debridement with subtotal left maxillectomy and reconstruction with a pedicled forearm flap	Alive
6(2021)**	Female	Han nationality	47	Type-2 diabetes mellitus (on irregular treatment with oral hypoglycemics)	No	Ketoacidosic diabetic coma; blindness in the left eye; swelling and tenderness of the left cheek; purulent nasal discharge; extensive ulceration and necrosis involved the orbital floor, the maxilla, the hard palate and alveolar process, resulting in exposed bone and exfoliation of the anterior teeth	WBC-16.90; NEUT%-86.00; neutrophils-14.53; Hct-35.5%; PLT-114	PCT-0.11; BUN-5.30; FBG-6.52; PPBS-33.35; Scr-104.40	A moth-eaten appearance of the left maxillary alveolar bone and haziness of the maxillary antrum of the same side	Intravenous liposomal AmB; Surgical debridement	Died*****
7(2021)***	Female	Han nationality	38	Severe aplastic anemia, pneumonia, hypoproteinemia, hypokalemia	NM	Fever; nasal sanguineous discharge; blackly nausea; exposed necrotic gray-colored bone in right maxillary molar region, with surrounding necrotic tissue	WBC-0.09; NEUT%-undetectable; neutrophils-undetectable; Hct-22.3%; PLT-7	IL 6-193.100; PCT-0.03; CRP- > 90.90; DD-2302.0; BUN-9.13; FBG-4.14; PPBS-21.89; Scr-27.01	A lesion of soft tissue density in the nasal cavity, causing destruction of the nasal septum centrally, destruction of the medial walls of the antra bilaterally and perforation of the palate inferiorly, partial destruction of the floor of the orbit and ethmoidal sinus	Refused surgical recommendations (debridement, definitive palatoplasty) and was transferred to ICU to continue antifungal therapy	Died******

AmB = amphotericin B. BUN = Blood urea nitrogen (mg/dL). CRP = C-reactive protein (mg/L). DD = D-dimer (ng/mL). ESR = Erythrocyte sedimentation rate (mm/1 h). FBG = Fasting blood glucose (mmol/L). HbA1c = glycohemoglobin. Hct = Hematocrit. ICU = Intensive care unit. IL = Interleukin (pg/mL). NA = Not available. NEUT% = Neutrophil percentage. NM = Not mentioned. PCT = Procalcitonin (ng/mL). PLT = Platelets (× 10^9^/L). PPBS = Post-prandial blood sugar level (mg/dL). Scr = Serum creatinine (mg/dL). WBC = White blood cell (× 10^9^/L)

*Outpatient transferred from our institute’s Department of Endocrinology

**Outpatient transferred from our institute’s Department of Emergency

***Inpatient transferred from our institute’s Department of Hematology

****Intravenous liposomal AmB (0.1 mg/kg increasing daily to 1 mg/kg, once a day) + oral Posaconazole (5 mL/2 mg, every 6 h)

*****Ultimately, the patient’s respiratory system was further compromised due to pneumonia, and she expired due to cardiopulmonary arrest 43 days after his surgical treatment

******Ultimately, the family decided to make the patient comfort care because of her poor prognosis. The patient expired on day 26 of her hospitalization

Prior to surgical operation, 6 of our patients underwent endocrine therapy to treat their primary illness; for the remaining aplastic anemia case, we planned to transfuse platelets during surgery, in cases where platelet count did not reach the minimum level (greater than 50,000). Antifungal pharmacologic (taking amphotericin B as the principal administration) and comprehensive surgical (partial or total maxillectomy, or inferior conchotomy and ostectomy of maxillary sinus) treatment were given to five patients (Table 1). And the surgeries were performed as soon as possible. The overall number of deaths was 5 (71.43%); nevertheless, only 1 patient (20%) was resulted from her primary disease and the other 4 patients’ deaths were caused by the disseminated fungal infections (80%).

## Literature review

**Fig. 3:**
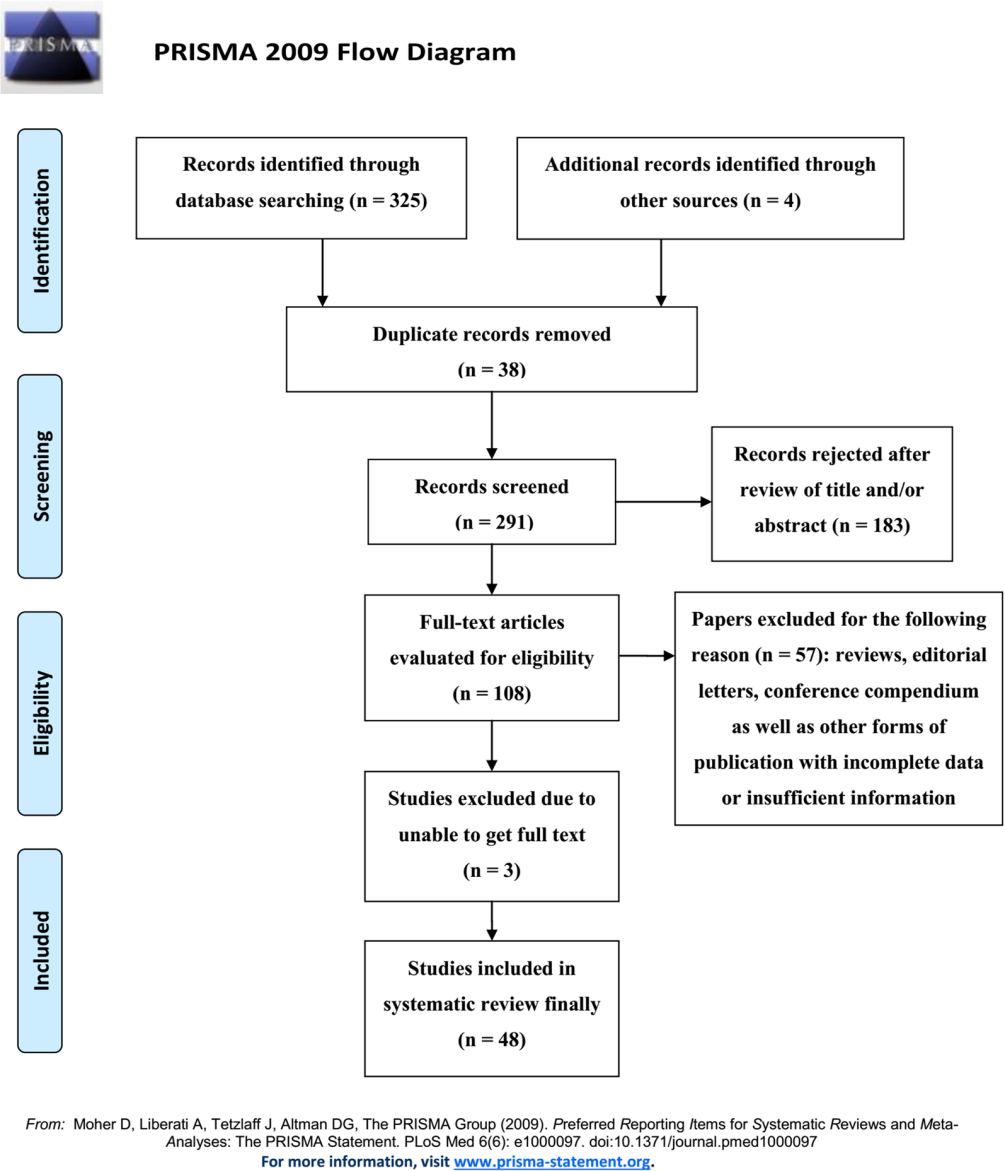
Flow diagram of the study selection process

The review section was registered on PROSPERO (International Prospective Register of Systematic Reviews) (CRD42022382257). We searched for relevant articles within several databanks (Pubmed, Embase, Web of Science, Ovid MEDLINE, and Scopus) published from database inception until June 2022. Search strings used were “mucormycosis”, “Zygomycetes”, “Mucorales”, “*Mucoromycotina*”, “fungal infections”, “head and neck region”, “maxillofacial” and “oral cavity”, in combination with Boolean Operators ‘OR’ and ‘AND’. We excluded studies lacking the necessary data and cases with other concomitant suppurative infection lesions resulted from bacteria, such as *Staphylococcus aureus*, *Streptococcus*, and *Escherichia coli*. The resulting publications were searched, and the references of all articles were verified to decrease the possibility of omitting relevant publications. The initial search and review of references retrieved 329 related articles, 183 of which were rejected because of information deficit after screening the titles and abstracts. Two experienced investigators (C. Li and Z. Gong) independently extracted the following characteristics from each eligible study: name of the first author, year of publication, geographical location, demographic information, scope of involvement, clinical manifestation, care given, follow-up period, and outcome (Table 2). The results of the systematic literature retrieval have been depicted in Fig. 3.

**Table 2:** Characteristics of studies recruited in the systematic review

Authors (time)	Country	Age (years)	Gender (F/M)	No. of patients	Predisposing diseases or risk factors	Involved sites/lesion	Laboratory test	Imaging findings	Management	Outcome
Eisenberg et al. [57]	USA	(1) 28 (2) 38 (3) 72	F	3	Corticosteroid (prednisone), acute renal failure (pyelonephritis), metabolic acidosis, hyperglycemia	(1) Mandible, maxillary alveolar ridges, nasal septum (2) Tongue, hard palate, uvula, eye (3) Cavernous sinus	(1) Hct-41, WBC-9,000, Scr-1.4, BUN-26, FBG-130 (2) Scr-2.8, BUN-63, FBG-260 (3) Hct-30, WBC-20,000, SMA-12	(1) Diffuse mucosal thickening in both maxillary sinuses and frontal sinuses (2) NM (3) Right cavernous sinus thrombosis	AmB, surgical debridement	Alive follow-up
Brown and Finn [56]	USA	57	M	1	Chronic renal failure, diabetes mellitus, hypertension	Mandible and adjacent soft tissue, lower lip	NM	Osteomyelitic mandible and a left parapharyngeal space abcess	AmB, mandibulectomy	Death—105 days
Jones et al. [55]	USA	(1) 43 (2) 68	M	2	(1) Acute myelogenous leukemia, acute renal failure, hepatosplenic candidiasis, pancytopenia (2) Diabetes mellitus (although well-controlled), persistent odontogenic infection, maxillary incisors extraction	(1) Right premolar region in the mandible, mandibular gingiva (2) Maxilla	NM	(1) Moderate horizontal and vertical bone loss (2) Fragments of necrotic bone	Fluconazole, AmB, surgical debridement	NM
Salisbury et al. [54]	USA	60	M	1	Acute myelogenous leukemia, prostate cancer, hypertension, heavy use of alcohol and tobacco, pancytopenia	Right mandibular second molar	WBC-1200/μL, neutrophils-200/μL, Hct-24.5%, platelets-15,000/μL	Deep vertical bone defects, periapical radiolucency	AmB, surgical debridement	Alive follow-up
Kim et al. [53]	USA	57	M	1	Insulin-dependent diabetes mellitus, diabetic ketoacidosis	Left cavernous sinus, left maxilla, maxillary, ethmoidal & frontal sinus, orbit, parotid, zygoma, supraorbital and frontal region	WBC-18,800/mm^3^, Hct-35.8%, platelets-201,000/cm^3^, Scr-1.5 mg/dL, glucose-428 mg/dL with positive ketones	Opacification of the left maxillary sinus, ethmoid sinus, and frontal sinus, early stage cavernous sinus thrombosis	Surgical debridement, frontal ventriculostomy (neurosurgical service)	Death – 7 days
Maiorano et al. [52]	Italy	66	M	1	Generalized Castleman disease, mild neutropenia	Right hemi-face, involving the peri-orbital, zygomatic and maxillary areas, naso-sinusal congestion and diffuse ulcerative lesions of the palatal and cheek mucosa	WBC-4900/mL, peripheral CD4 T cells- > 500/μL	A soft tissue tumour-like expansion with central necrosis, involving the palatal mucosa and the paranasal sinuses	AmB, surgical debridement	Alive follow-up (3.5 years)
Fogarty et al. [51]	Canada	74	M	1	COPD, corticosteroid, dental extraction	Left maxilla, inferior zygoma, nasal septum, pterygoid plate	NM	somewhat radiolucent of the contours of the lamina durae and sinus floor; lytic, destructive bony process	Low level maxillectomy, liposomal AmB	Death—43 days
Alfano et al. [50]	Italy	50	F	1	Ketoacidosic diabetic coma, periodontitis, dental extraction, cerebral ischemia	Orbital floor, maxilla, hard palate, alveolar process	WBC-8,800 μL^−1^, glucose-580 mg dL^−1^, pH-7.09	Variable clouding of the paranasal sinuses and destruction of the anterior sphenoidal wall	Aggressive surgical debridement; Specific antifungal regimen, consisting of *i.v.* combined therapy of Voriconazole (6 mg/kg for 2 doses, followed by 4 mg/kg twice a day), Caspofungin (70 mg on day 1, followed by 50 mg/day once a day), and liposomal AmB (1.5 mg/kg/day once a day)	Alive follow-up (16 months)
Lador et al. [49]	Israel	42	F	1	Acute lymphoblastic leukemia, pancytopenia	a broad necrotic ulcer extending from the lingual aspect of teeth 21 to 25 to the adjacent labial mucosa	WBC-200/μL, platelets-6,000/μL	Normal mandible	AmB, surgical debridement	Death—14 days
Pellacchia [48]	Italy	57	F	1	Diabetes mellitus	Para nasal sinus and brain	NA	Cerebral abscess	Surgical resection of the ethmoid-spheno-maxillo-orbital district	NA
Jayachandran and Krithika [47]	India	48	M	1	Poor diabetic status, glycosuria, ketonuria, polymorphonuclear leucocytosis	Nasal cavity, left lower eyelid and infra orbital sinus, palate	FBG-426 mg/dL	Destruction of the nasal septum centrally, destruction of the medial walls of the antra bilaterally and perforation of the palate inferiorly	AmB, cefotaxime and metronidazole surgical debridement	Alive follow-up
Bakathir [46]	Oman	(1) 14 (2) 49	M	2	(1) Acute myeloid leukaemia, dental extraction (2) Type-2 diabetes mellitus, diabetic ketoacidosis, neutropenia, pancytopenia, acute lymphoblastic leukaemia	(1) Right maxillary sinus, nose and ethmoid; mandible alveolar bone (2) Mandible	(1) Platelets-38 × 10^9^ (2) WBC-1.52 × 10^9^/L, platelets-44.2 × 10^9^, glucose-21 mmol/L	(1) A soft tissue swelling of the right infra-orbital region and marked soft tissue obliteration of the right maxillary sinus (2) Bone destruction	(1) Surgical debridement twice (with partial maxillectomy and FEES), irrigation with 0.2% Chlorhexidine antiseptic mouth wash, 3% hydrogen peroxide solutions (2) AmB, surgical debridement	Alive follow-up
Dogan et al. [45]	Turkey	(1) 7 (2) 9	M	2	(1) Acute myeloid leukemia (2) Acute lymphoblastic leukemia	Intraoral soft tissue lesions	NA	NA	(1) AmB, surgical debridement (2) Antifungal therapy	NM
Auluck [44]	India	58	M	1	Diabetics, poor periodontal health	Maxillary alveolus and right maxillary sinus	FBG-238 mg/dL, post prandial blood sugar-386 mg/dL	Haziness of right maxillary sinus with erosion of lateral sinus wall	AmB, surgical debridement	Alive follow-up
Kishel and Sivik [42]	USA	49	F	1	Acute myeloid leukemia	Pansinusitis	NM	NM	AmB lipid	Death—7 days
Chua and Cullen [43]	Singapore	(1) 41 (2) 56	1/1	2	Poorly controlled diabetes mellitus, hyperosmolar non-ketotic acidosis	Orbit and left maxilla, bilateral ethmoid sinus, and sphenoid sinus	(1) HbA1c-10.7% (2) HbA1c-12.8%	(1) Pan-sinusitis, involving the left maxillary, bilateral ethmoid and sphenoid sinuses, with involvement of the left orbital apex (2) Pan-sinusitis, involving bilateral sphenoid, left ethmoid (anterior and posterior) and left maxillary sinuses, with obstruction of the left ostiomeatal unit and spheno-ethmoidal recess	(1) AmB, flagyl, augmentin; surgical debridement and septoplasty, FEES, left ethmoidectomy (2) AmB, flagyl, augmentin; surgical debridement and left uncinectomy, anterior and posterior ethmoidectomy as well as sphenoidectomy	Alive follow-up 1. Light perception with consequent optic atrophy; 2. No light perception of vision, third and trigeminal nerve palsies)
McDermott et al. [41]	USA	47	F	1	Myelodysplastic syndrome, acute myelogenous leukemia	Left maxilla	WBC-60 cells/μL (0.06 K/μL), absolute neutrophil-undetected (< 0.00 K/μL), Hct-31.2%, platelets-20 K/μL	Negative for infectious etiology, no evidence of bone destruction	Surgical debridement, T-cell–depleted allogeneic stem cell transplant	Alive follow-up
Ojeda-Uribe et al. [40]	France	55	F	1	Acute myeloid leukaemia, decompensated diabetes mellitus, neutropenic	Lower lip, chin, floor of the mouth, a portion of the tongue, as well as mandible	Neutrophils-0.1 × 10^9^/L	Necrotic areas in the base of the oral cavity, pre-mandibular soft tissue and in a portion of the mobile tongue, but without involvement of the mandibula	AmB, Caspofungin, surgical debridement, mandibulectomy	Alive follow-up (6 years)
Papadogeorgakis et al. [39]	Greece	22	F	1	Type I diabetes mellitus, dental extraction	Paranasal sinus on right side	HbA1c-12.3%, FBG-197 mg/dL, WBC-9.47 × 10^3^, CRP-20.01 mg/dL	Thickening of the mucosal lining of the paranasal sinuses	AmB, posaconazole, subtotal right maxillectomy followed by obturator	Alive follow-up
Pandey et al. [38]	India	(1) 42 (2) 62 (3) 65 (4) 70	3/1	4	(1) Uncontrolled diabetes mellitus, self-extraction (2) Uncontrolled diabetes mellitus, self-extraction (3) Uncontrolled diabetes mellitus, self-extraction (4) Uncontrolled diabetes mellitus on renal dialysis, chemical injury	Upper jaw	1), 2), (4) NM (3) FBG-230 mg/dL, post prandial blood sugar-356 mg/dL	(1) NM (2) Maxillary sinus opacification with posterior wall destruction (3) Radio-opaque maxillary antrum and posterior wall destruction (4) Typical maxillary sinus opacification with sinus wall destruction	(1) Complete debridement of maxillary antrum through Caldwell–Luc procedure; inferior meatus antrostomy and buccal advancement flap for closure of oro-antral fistula. Liposomal AmB (2) Extraction of upper teeth, sequestrectomy and debridement of maxillary antrum. Liposomal AmB (3) Maxillary sequestrectomy, total antral curettage and complete debridement. Liposomal AmB (4) Local debridement. Ketoconazole	(1) Satisfactory on follow-up to 16 months (2) Satisfactory on follow-up to 24 months (3) Satisfactory on follow-up to 24 months (4) Satisfactory on follow-up to 18 months
Doni et al. [37]	India	49	M	1	Chronic renal failure, hypertension, type II diabetes mellitus, polymorphonuclear leukocytosis	Rhino-maxillary	WBC-12,000/mm^3^, ESR-40 mm/1 h, random blood sugar-316 mg/dL, Scr-3.7 mg/dL	Soft tissue density with sclerosis of bony wall in relation to right maxillary sinus and defect in maxillary bone in the floor of right maxillary sinus	AmB, surgical debridement	Death—30 weeks
Vaidya and Shah [36]	India	68	M	1	Hypertension, cardiovascular stroke	Right posterior ethmoid, sphenoid, and maxillary sinuses	WBC-15,800/cumm, high protein count of 75 mg/dL detected in cerebrospinal fluid	Mucosal thickening of all sinuses with cellulitic changes in right orbit involving extraconal, intraconal, and preseptal compartment of orbit	Removal of fungal debris along with necrotic tissues by endoscopic surgery	NM
Suwan et al. [35]	Thailand	NA	NA	2	Case 1: T-cell lymphoma Case 2: Tetralogy of fallot	Case 1: Left maxillary, frontal, ethmoid Case 2: Left maxillary and ethmoid sinus	NA	NA	NA	NA
Aras et al. [34]	Turkey	(1) 6 (2) 15	M	2	(1) Neuroblastoma (2) Acute myelogenous leukemia, thrombocytopenia	(1) Right mandibula (2) Left mandibula	(1) WBC-5.84 × 10^3^μL, absolute neutrophil-2.5 × 10^3^μL, Hct-31.4%, platelets-423 × 10^3^μL (2) WBC-247 × 10^3^μL, absolute neutrophil-57.4 × 10^3^μL, Hct-26.2%, platelets-109 × 10^3^μL	(1) NM (2) Left mandibular premolar and molar region was encompassed by white necrotic appearing tissue	AmB, surgical debridement	(1) Death—3 months (2) Death—7 months
Ananthaneni et al. [33]	India	63	M	1	Diabetics (20 years), long-term medication of aspirin (5 years)	Upper back jaw region	FBG-90 mg/dL	Destruction of the anterior hard palate and maxillary alveolar process from 21 to 18	Right maxillectomy; flucanazole 150 mg orally for 6 months, and a combination drug of cefoperazone and sulbactum 1.2 g intravenously for 10 days, both of which were given twice daily and metronidazole 100 ml intravenously thrice daily	Alive follow-up (12 months)
Kumar et al. [32]	India	65	M	1	Diabetics (10 years) on irregular treatment with oral hypoglycemics, dental extraction	Entire maxillary alveolar process of palate up to the soft palate region	NM	Erosion of the anterolateral wall of right maxillary antrum with thickening of the sinus lining with moth eaten appearance of the maxillary bone seen	NM	NA
Garlapati et al. [30]	India	40	F	1	Uncontrolled type II diabetes mellitus	Maxilla, paranasal sinuses	FBG-300 mg/dL, post prandial blood sugar-402 mg/dL	Nonhomogenous opacification of left maxillary sinus causing obstruction of left osteomeatal unit extending into middle meatus, ethmoidal, and frontal sinus causing destruction of walls of left maxillary and ethmoidal sinuses	AmB, surgical debridement	Satisfactory in follow-up
Choudhary et al. [31]	India	48	F	1	Non immuno-compromised, dental extraction	Maxillary alveolus, palate, left maxillary sinus and nose	NA	NA	NA	NA
Motaleb et al. [27]	Egypt	57	F	1	Facial cellulitis, dental extraction	Right buccal, infraorbital and temporal areas, chemosis	Absolute neutrophil-27.47 × 10^3^, BUN-32.0 mg/dL, Scr-1.40 mg/dL, Hct-29.1%, platelets-242 × 10^3^, random blood glucose-158 mg/dL	Clouding of the right nasal cavity, maxillary, ethmoid, frontal and sphenoid sinuses	AmB, surgical debridement	Death—14 days
Nilesh et al. [26]	India	72	M	1	No significant medical or family history was reported. Dental extraction	A partially edentulous maxillary arch (left)	NA	Thickening of the left maxillary sinus lining	Surgical debridement, Posaconazole (400 mg twice daily for 6 weeks)	Satisfactory on follow-up to 6 months
Kumar et al. [25]	India	63	F	1	Type-II diabetes mellitus, dental extraction	Upper jaw (left maxillary sinus and maxilla)	Random blood sugar-346 mg/dL, FBG-136 mg/dL, post prandial blood sugar-226 mg/dL	Bone destruction in anterior maxillary wall with obliteration of left maxillary sinus	AmB, surgical debridement	Alive follow-up
Selvamani et al. [24]	India	52	M	1	Type II diabetes mellitus, dental extraction	Right maxillary sinus and anterior palatal region	NM	Haziness in the right maxillary region and perforation in the anterior palatal region	AmB, surgical debridement with anterior maxillectomy	NM
Arya et al. [29]	India	54	M	1	Diabetes Mellitus, dental extraction	Bilateral maxillary and sphenoid sinuses, right maxilla, bilateral nasal cavity, ethmoidal air cells and pterygoid	NA	NA	Surgical debridement with maxillary obturator, AmB, voriconazole	NA
Fanny et al. [28]	Indonesia	46	F	1	Non immuno-compromised, dental extraction	NM	NM	NM	Hyperbaric oxygen	Alive follow-up
Mahomed et al. [23]	South Africa	54	F	1	Insulin-dependent diabetes mellitus, diabetic ketoacidotic coma with peri-orbital cellulitis and neurological impairment	Right orbit, right maxillary sinus, bilateral ethmoid, right sphenoid sinus and cavernous sinus	Glucose level-mmol/L, CRP-38, 1 + ketones present on urine dipstick testing	Partial opacification of the right maxillary, bilateral ethmoid and right sphenoid sinuses	Endoscopic antrostomy for debridement of the necrotic tissue, as well as an external fronto-ethmoidectomy; AmB	Alive follow-up (6 weeks) (irreversible blindness in the right eye as well as cranial nerve palsies)
Habroosh et al. [22]	United Arab Emirates	17 months	M	1	Long term of intermittent systemic antibiotics due to diagnosis of dacrocystitis	Right medial canthal	NM	Right superior medial upper lid along the medial canthal area and extending inferiorly to the medial inferior orbital rim	AmB and Voriconazole; surgical debridement	NA
Afroze et al. [21]	India	50	F	1	Uncontrolled diabetes, asthmatic, dental extraction	Right maxilla	FBG-154 mg/dL, post prandial blood sugar-197 mg/dL, HbA1c-8.3%	Hyperdensity of the maxillary antrum with destruction of all the boundaries of sinus including nasal wall and floor of the orbit	Venofer, surgical debridement	Alive follow-up (1 year)
Nilesh and Vande [20]	India	(1) 37 (2) 52	M	2	Non immuno-compromised, dental extraction	(1) Maxillary alveolar bone (2) Left maxilla and maxillary sinus	NA	(1) Closure of the surgical site after removal of the necrosed alveolar bone (2) Thickening of left maxillary antrum lining, with destruction of anterior maxillary wall	AmB, surgical debridement	(1) Satisfactory on follow-up to 4 months (2) Satisfactory on follow-up to 6 months
Prabhu et al. [19]	Bahrain	70	M	1	Uncontrolled diabetes mellitus, dental extraction	Bilaterally throughout the nasal mucosa; right hard palate, alveolar ridge, and buccal mucosa	NM	Heterogeneous opacity in left maxillary sinus	Liposomal AmB; surgical debridement with right hemimaxillectomy, turbinectomy, uncinectomy, middle meatal antrostomy, ethmoidectomy and sphenoidectomy	Death—10 days
Gholinejad Ghadi et al. [18]	Iran	(1) 36 (2) 53	1/1	2	(1) Uncontrolled diabetes mellitus, dental extraction (2) Uncontrolled diabetes mellitus, dental extraction, neutropenia,	(1) Left posterior maxilla, paranasal sinuses (2) Left posterior maxilla, the corresponding gum	(1) FBG-231 mg/dL, 2 h postprandial blood sugar-248 mg/dL (2) FBG-253 mg/dL, 2 h postprandial blood sugar-480 mg/dL,	(1) Typical maxillary sinus opacification with sinus wall erosion and thickening (2) Paranasal, maxillary and ethmoid sinuses showed opacity and posterior wall destruction	(1) AmB, posaconazole; surgical debridement and FEES (2) AmB, surgical debridement, FEES, maxillary sequestrectomy	(1) Satisfactory on follow-up to 56 days (2) NA
Arani et al. [17]	Saudi Arabia	48	M	1	Diabetics, dental extraction	Left posterior back tooth region of the upper jaw	NM	Radiolucency extending from the alveolar ridge to maxillary sinus, breaking the floor of the sinus in relation to 26	Surgical debridement	NM
Srivastava et al. [16]	India	62	F	1	Type II diabetes mellitus, and the exodontia	Left midfacial region	FBG-139 mg/dL, post prandial blood sugar-193 mg/dL	A moth-eaten appearance of the left maxillary alveolar bone and haziness of the maxillary antrum of the same side	AmB, voriconazole; surgical debridement	Alive follow-up (2 years)
Mehta and Pandey [15]	India	60	M	1	Longstanding diabetics (> 10 years), mild lymphopenia, hypotension, COVID-19	Right rhino-orbital	Scr-1.57 mg/dL, CRP-29.53 mg/L, PCT-0.34 ng/mL, D-dimer assay of 1547 ng/mL, IL6 level of 3439 µg/mL	NM	Meropenem, vancomycin, AmB	Death—6 days
Dallalzadeh et al. [14]	USA	(1) 36 (2) 48	M	2	Type 2 diabetes, COVID-19	Rhino-orbital cerebral	Sars-CoV-2 positive	(1) Flow void through the left ophthalmic artery and superior ophthalmic vein; absence of enhancement within the left medial and lateral rectus muscles and along the optic nerve/sheath (2) The right sinonasal cavity and anterior skull base extending to the bilateral frontal lobes	(1) Urgent lateral canthotomy and cantholysis; AmB, isovuconazole, and micafungin; the cessation of corticosteroids (2) AmB, isovuconazole; surgical debridement was deferred	(1) Death—4 days (2) NM
Mekonnen et al. [13]	USA	60	M	1	Poorly controlled insulin-dependent diabetes, asthma, hypertension, hyperlipidemia, bronchitis	Right rhino-orbital	HbA1c-14.0%, serum glucose was mildly elevated (105–143 mg/dL), Sars-CoV-2 positive	Right globe proptosis with asymmetric retrobulbar fat stranding and extensive opacification of right maxillary, ethmoid, and frontal sinuses; partial opacification of the right sphenoid sinus and erosions of the lamina papyracea	AmB, caspofungin; surgical debridement	Death—31 days
Werthman-Ehrenreich [12]	USA	33	F	1	Hypertension, asthma, mild tachycardia, tachypnea	Left eye ptosis with 1 cm proptosis	WBC count of 27 with 82.9% neutrophils and 5.1% lymphocytes; glucose 649, Scr 2.28, and lactate 2.8; Sars-CoV-2 positive	Bilateral maxillary sinus mucosal thickening as well as ethmoid sinus mucosal thickening, and mucosal opacification of the ostiomeatal units	AmB; Neurosurgery was consulted for possible operative intervention but declined because of poor prognosis	Death—26 days
Emodi et al. [11]	Israel	(1) 19 (2) 22 (3) 23 (4) 24 (5) 27 (6) 30 (7) 42 (8) 48 (9) 56 (10) 67	(1) F (2) M (3) F (4) M (5) F (6) F (7) F (8) M (9) F (10) F	10	(1) Burkitt leukemia (2) Chronic myeloid leukemia; maxillary exodontia; multiple extractions in mandible (3) Acute myeloid leukemia; maxillary exodontia (4) Acute lymphoblastic leukemia (5) Acute myeloid leukemia (6) Acute myeloid leukemia; maxillary exodontia (7) Acute myeloid leukemia; maxillary exodontia (8) T-cell acute lymphoblastic leukemia; maxillary exodontia (9) Diffuse mixed cell lymphoma (10) Acute myeloid leukemia	(1) Escape of fluid through nose, black lesion on the right nostril and hard palate (2) Surroundings of extraction wound with necrosis (3) Ulcer on hard palate (4) Pain in palpation left maxilla (5) Right maxillary swelling, vestibular swelling, pain and necrosis of gum (6) Nonhealing extraction site with a massive hematoma in maxillary anterior right area (7) Nonhealing extraction site with necrosis of alveolar bone and palate (8) Nonhealing extraction site with black lesion, nasal congestion in maxillary anterior right area (9) Hard palate necrosis (10) Painful swelling of right face, palatal necrosis, fever	(1)WBC-1.9 × 10^3^, Neutrophils-1.06 × 10^3^, PLT-77 × 10^3^, Blood Glucose-97 mg/dL (2)WBC-0.7 × 10^3^, Neutrophils-0.6 × 10^3^, PLT-52 × 10^3^, Blood Glucose-132 mg/dL (3) WBC-0.8 × 10^3^, Neutrophils-0.4 × 10^3^, PLT-190 × 10^3^, Blood Glucose-143 mg/dL (4) WBC-5.4 × 10^3^, Neutrophils-3.4 × 10^3^, PLT-144 × 10^3^, Blood Glucose-122 mg/dL (5) WBC-2.4 × 10^3^, Neutrophils-2.4 × 10^3^, PLT-77 × 10^3^, Blood Glucose-138 mg/dL (6) WBC-0.5 × 10^3^, Neutrophils-0.01 × 10^3^, PLT-3 × 10^3^, Blood Glucose-100 mg/dL (7) WBC-1.7 × 10^3^, Neutrophils-0.9 × 10^3^, PLT-35 × 10^3^, Blood Glucose-131 mg/dL (8) WBC-12.7 × 10^3^, Neutrophils-4.3 × 10^3^, PLT-18 × 10^3^, Blood Glucose-122 mg/dL 9) WBC-0.5 × 10^3^, Neutrophils-0.01 × 10^3^, PLT-10 × 10^3^, Blood Glucose-138 mg/dL 10) WBC-0.5 × 10^3^, Neutrophils-0.01 × 10^3^, PLT-18 × 10^3^, Blood Glucose-124 mg/dL	(1) Nasal mucosa thickening (2) Right maxillary sinus fullness, socket nonhealing (3) Left maxillary sinus thickening (4) Left maxillary sinus fullness (5) Left maxillary sinus thickening (6) Right maxillary sinus thickening (7) Anterior socket nonhealing, nasal mucosa thickening (8) Nasal mucosa thickening (9) Left maxillary sinus thickening (10) Right maxillary sinus thickening	(1) AmB; Removal of right nostril inferior concha, partial ostectomy of maxillary sinus (2) AmB; Partial maxillectomy (3) AmB; Subtotal maxillectomy (4) AmB; Partial maxillectomy (5) AmB; Surgical debridement with partial maxillectomy (6) AmB; Surgical debridement with partial maxillectomy (7) AmB; Surgical debridement with anterior maxillectomy (8) AmB; Surgical debridement with partial maxillectomy (9) AmB; Total maxillectomy (10) AmB; Hemimaxillectomy	(1) Death—24 days (2) Death—272 days (3) Alive follow-up (4) Alive follow-up (5) Death—81 days (6) Alive follow-up (7) Death—58 days (8) Death—28 days (9) Death—20 days (10) Death—288 days
Moorthy et al. [10]	India	Mean aged 54.67 (35–73 years old)	3/15	18	SARS-CoV-2; Uncontrolled Diabetes and Corticosteroids	Maxillofacial/rhino-cerebro-orbital	NM	NA	Liposomal AmB; Surgical debridement assisted with sinus endoscopy	Survival rate 66.67% (n = 12)

*AmB* amphotericin B, *BUN* blood urea nitrogen, *COPD* Chronic obstructive pulmonary disease, *COVID-19* Corona virus disease 2019, *CRP* C-reactive protein, *ESR* erythrocyte sedimentation rate, *F* female, *FEES* functional endoscopic sinus surgery, *FBG* fasting blood glucose, *HbA1c* glycohemoglobin, *Hct* hematocrit, *IL* interleukin, *M* male, *NA* not available, *NM* not mentioned, *PCT* procalcitonin, *PLT* platelets, *Scr* serum creatinine, *SMA* smooth muscle autoantibody, *WBC* white blood cell

**Fig. 4:**
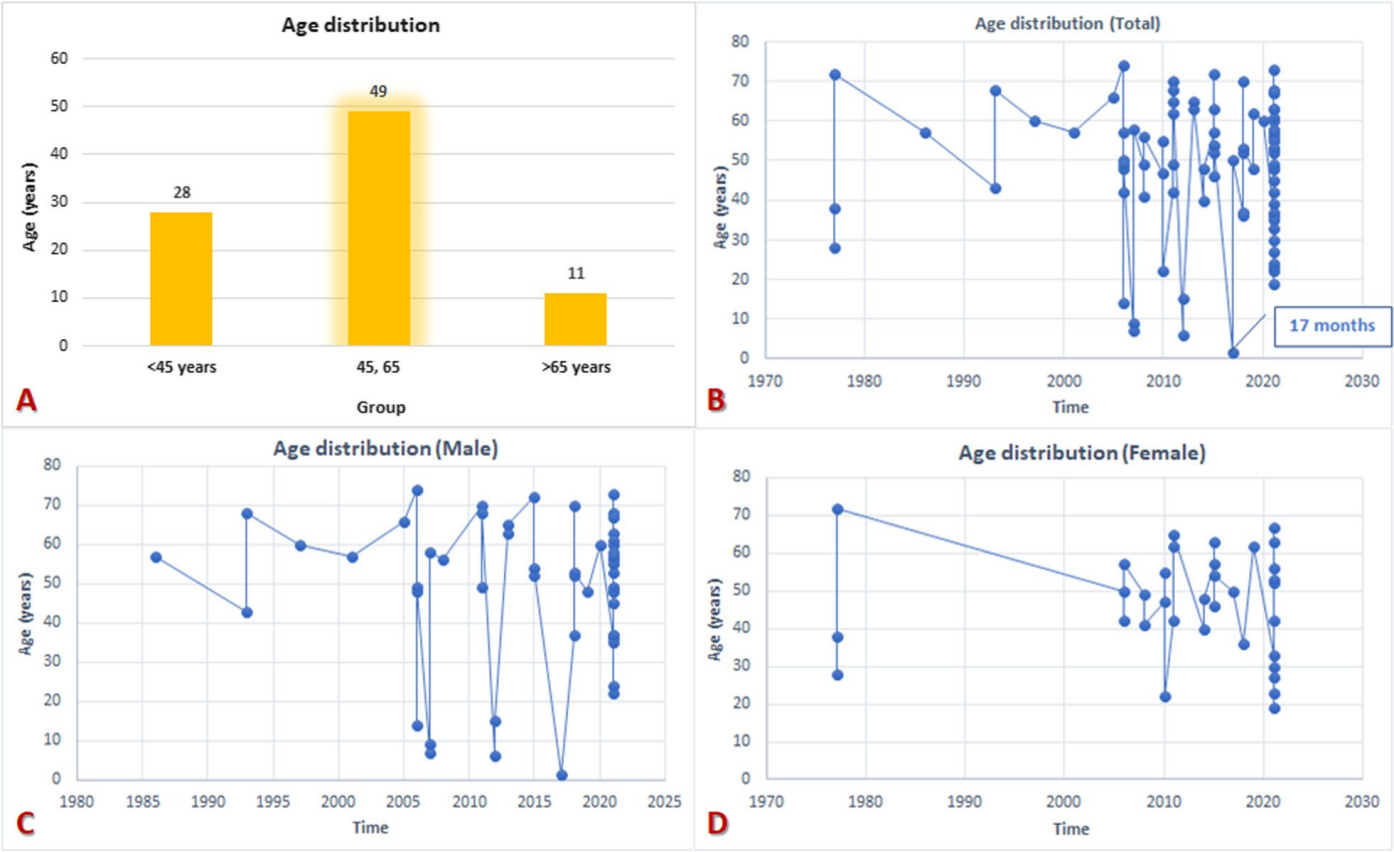
Characteristics of the age distribution reviewing of all extant literature of mucormycosis in cranio-maxillo-facial region. **A** grouping by the frequency; **B** general trend; **C** male trend; **D** female trend

**Fig. 5:**
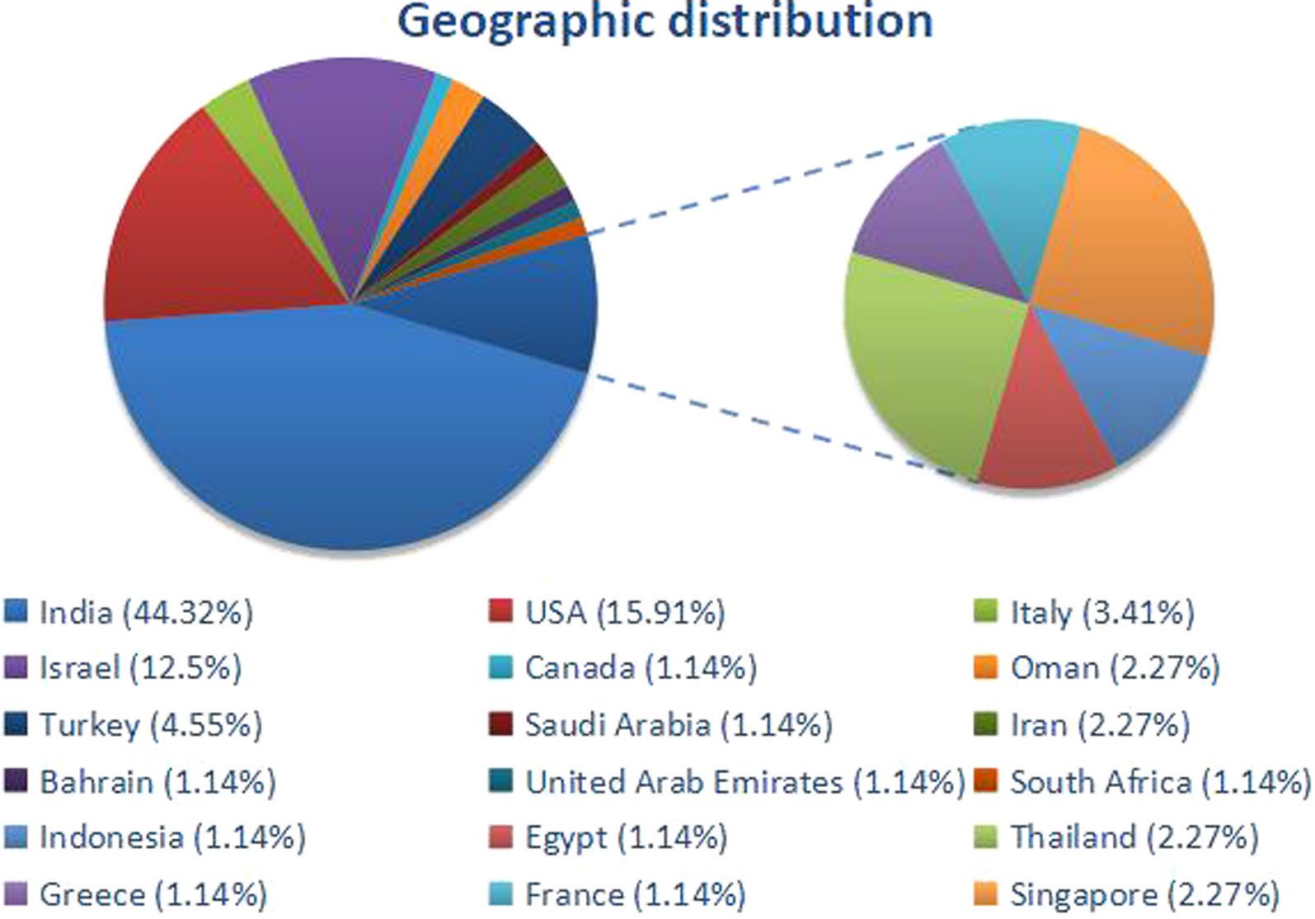
Distribution of all the cases of cranio-maxillo-facial mucormycosis that was reported from all over the world

We included 48 articles [10–57] with available and suitable data, contributing to the craniomaxillofacial mucormycosis. A total of 88 patients suffered from mucormycosis, of whom 28 (31.82%) were secondary to tooth extraction. The majority of patients were aged 45 to 65 years (55.68%, n = 49), and males were a little more than females with a sex ratio of 1.59:1 (*M*_n_ = 54, *F*_n_ = 34) (Fig. 4). These cases have been reported from across the world, among them, 39 cases (44.32%) from India; 14 (15.91%) from the United States; 11(12.50%) from Israel; 4 (4.55%) from Turkey; 3 (3.41%) from Italy; 2 (2.27%) from Thailand, Singapore, Iran and Oman; 1 case each (1.14%) from Canada, Saudi Arabia, Bahrain, United Arab Emirates, South Africa, Indonesia, Egypt, Greece and France (Fig. 5). The fundamental conditions predisposing mucormycosis involved in cranio-maxillo-facial region were as follows: a. diabetes mellitus with or without ketoacidosis (25 cases, 28.41 percent); b. acute/chronic myeloid leukemia (12 cases, 13.64 percent); c. acute lymphoblastic leukemia (4 cases, 4.55 percent); d. diabetes along with renal failure/hypertension (5 cases, 5.68 percentage); e. diabetics along with leukemia/neutropenia (3 cases, 3.41 percentage); f. diabetes along with asthma/hypertension (3 cases, 3.41 percentage); g. lymphoma (2 cases, 2.27 percentage); h. leukemia along with renal failure, i. generalized Castleman disease, j. chronic obstructive pulmonary disease treated with corticosteroids, k. cardiovascular stroke, l. tetralogy of fallot, m. neuroblastoma, n. long-term of intermittent systemic antibiotics, o. Burkitt leukemia (1 case, 1.14 percentage); notably, as well as p. diabetes mellitus along with COVID-19 (20 cases, 22.73 percentage). But, there also exited non-immunocompromised patients (6 cases, 6.82 percentage) suffering with this fungal infection (Table 2).

## Discussion

Mucormycosis, as a rare and fatal deep fungal infection, is given rise to the family Mucoraceae (Mucorales order, in detail, the *Absidia*, *Mucor*, *Rhizomucor*, *Rhizopus* genera can be the most frequently isolated strain from infecting patients) [58, 59]. These fungi are recognized as filamentous, ferrophillic, saprophytic, and ubiquitous in the nature, collectively known as phycomycetes [60]. Changes in molecular taxonomy and nomenclature support and decide the use of the appropriate name mucormycosis instead of the broader name zygomycosis, which describes any invasive fungal infection caused by the former Zygomycota phylum species [61, 62].

Due to its scarcity, this is always being a formidable challenge for maxillofacial clinicians to work on the diagnosis of mucormycosis, which is also the key crux to the entire therapeutic schedule. Based on the microbiological characteristics of mucormycosis, the experience of our clinical practice as well as the information of our critical review, we reckon that some points deserve attention as the following sections.

**Fig. 6:**
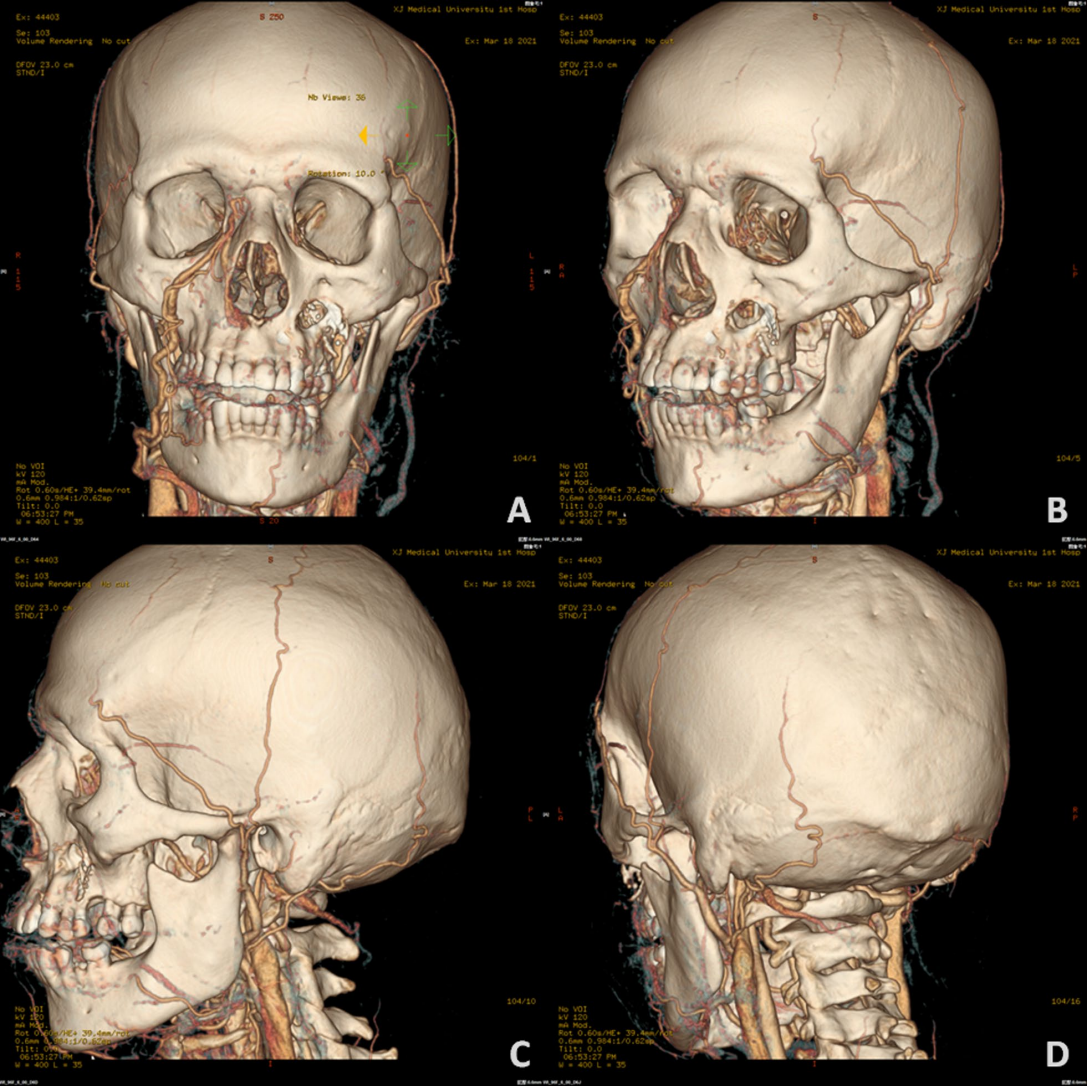
The computerized tomography angiography and three-dimensional reconstruction showed that left facial artery and ophthalmic artery were not developed indicating thromboembolisms existence. **A** Frontal view; **B** Left 45-degree-angled view; **C** Left side view; **D** Left posterior view

(1) *Disease site.* Generally, mucormycosis can be seen in pulmonary, cutaneous, rhino-orbito-cerebral, and even gastrointestinal infections [63–66]. Among them, rhino-orbito-cerebral fungus is the most prevalent form of mucormycosis, representing around 1/3–1/2 of all cases, of which approximately 90% occurring in head and neck region is affected by *Rhizopus* [67]. Maxilla, particularly the four pairs of sinuses (i.e. frontal, sphenoid, ethmoid, maxillary) [68], makes an ideal niche, wherein the stable habitat of constant temperature and humidity provided for the growth of these fungi which are also usual commensalism of nasal mucosa. Hence, the early stage of maxillofacial mucormycosis can be masqueraded as osteomyelitis and maxillary sinusitis that mimicking pain or toothache (seen in all our patients). In this stage, presentation include a potential onset of sinus drainage, sinus pressure, and soft tissue swelling, while it may be increasingly progressive and diffuse to adjacent tissues.

(2) *Typical clinical appearance*. Grey-black discoloration is observed when the involved tissues undergo necrosis resulting from thrombosed blood vessels. In particular, when the embolization of maxillary, facial or ophthalmic artery forms, massive necrotic area that donated by them can be seen through physical examination. Accordingly, CT angiography may be considered as a necessary imaging examination for maxillofacial mucormycosis (Fig. 6). Proptosis and ophthalmoplegia (restricted motion of eyeball) and loss of vision are often resulted from extension into the periorbital region and eventually into the orbit/globe [44, 69]. Mucosa-lined, air-filled sinuses promote the invasion into oral cavity giving rise to painful, necrotizing ulcerations usually with a blackish slough [70]. Through intraoral examination, it can be shown the exposure of black bare bone with the deprivation of mucoperiosteum over the entire maxillary alveolar process and hard palate up to the soft palate, sometimes occurring palatal fistula in the soft palate area [71].

(3) *Predisposing illness or risk factors*. Organ systems as well as microorganisms involved in the location of contamination associate closely with the clinical manifestations of mucormycosis. Maxillofacial mucormycosis is typically caused by direct infection of non-healing/incurable wound or inhalation/swallow of fungal spores. By reviewing the literature, we found mucormycosis affecting maxillofacial region predominantly appeared in immunocompromised patients (n = 84, 95.45%), embracing diabetes mellitus, hematological disorders, malignant tumor, etc. In our case series presenting maxillofacial mucormycosis, primarily diabetics (n = 6, 85.71%) had been identified likewise. On the other side, mucormycosis is capable to afflict healthy individuals owing to the role of local factors in the pathogenesis of this disease as described by Mignogna et al. [72] Local risk factors for example surgical trauma of a tooth extraction could damnify the local vascularity, thereupon then it would provide the microbes with a portal of entry. As per the reviewed literature, nonetheless, merely 5.68% (5/88) mucormycosis patients who had no significant medical or family history were secondary to the exodontia. Additionally, even though 31.82% (28/88) individuals demonstrated their mucormycosis suffering was next to a history of dental extraction, these patients were totally on the basis of dysimmunity of body condition. Furthermore, it is well-known that hyperglycemic hyperosmolar status, low pH value, and iron-rich environment in abnormal immune metabolism favors fungous growths [73] (Additional file 2: Fig. S1). These findings are consistent with the fact that oromaxillofacial invasive mucormycosis attacks mostly patients with compromised immunity (e.g. in our cases, 100%, presented in Table 1).

**Fig. 7:**
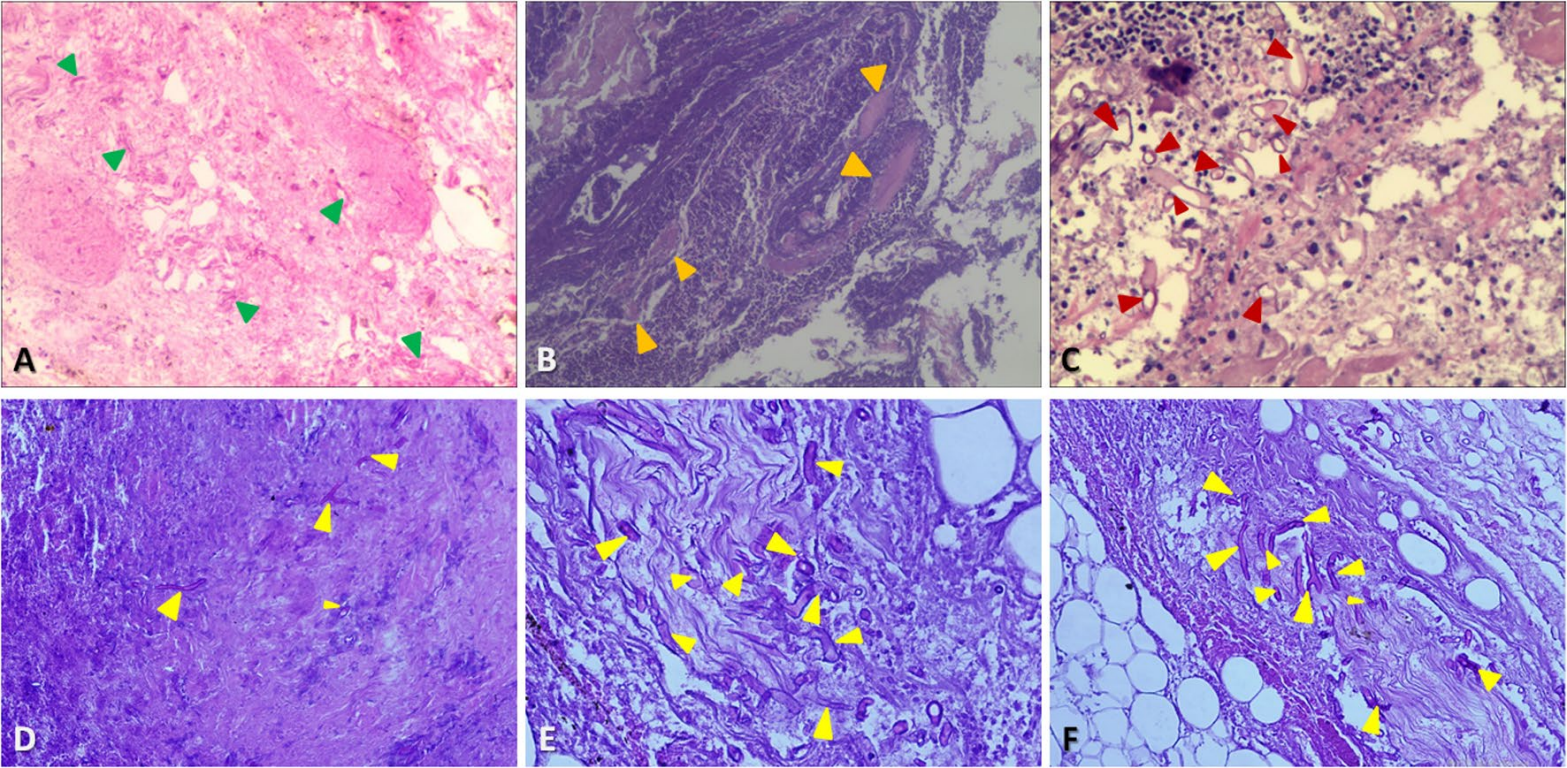
Histopathological examination of mucormycosis (microscopic view). **A** Bone and sinus tissue (100× magnification, H&E*): Area of acute inflammation and fungal forms infiltration arteries (arrow); **B** Sinus specimen (400× magnification, H&E*): Colonies of fungal spores and non-septate hyphae invaded the lumen of a blood vessel, causing thrombosis (arrow); **C** Sinus tissue (400× magnification, PAS**): Multinucleated giant cells with a numerous PAS-positive fragments in the cytoplasm (arrow); **D** Sinus section (100× magnification, GMS***): Intravascular invasion by fungal hyphae (arrow); **E** Sinus tissue (400× magnification, PAS**): Non septate wide branched hyphae (arrow); **F** Sinus tissue (400× magnification, PAS**): Numerous fungal hyphae which were aseptate, broad with obtuse angle branching, and without spore formation (arrow). *H&E = Hematoxylin and Eosin stain. **PAS = Periodic acid-Schiff stain. ***GMS = Gomori methenamine-silver stain

**Fig. 8:**
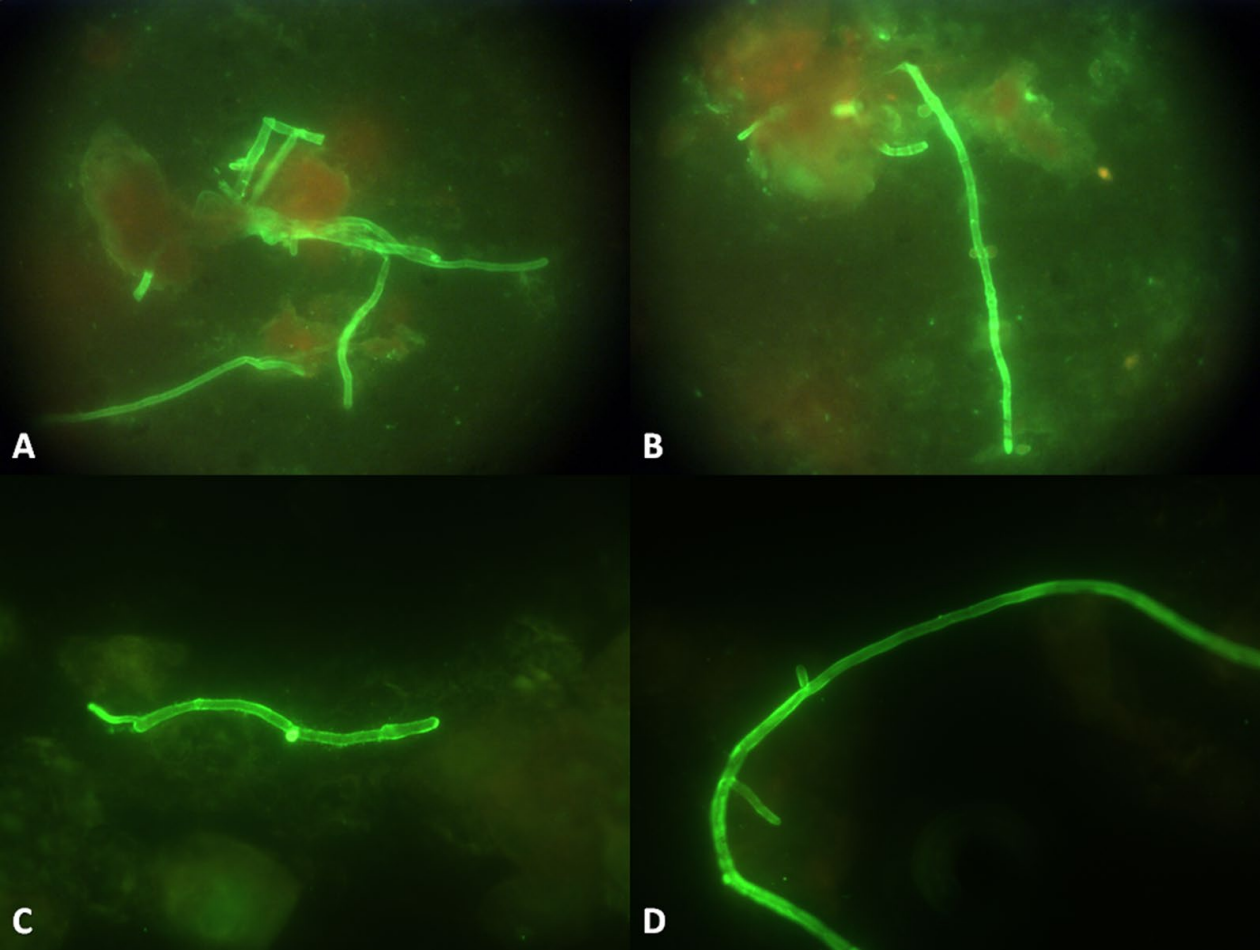
Sizes and branching angles for Mucorales and aspergillus were stained by fluorescence. **A** and **B** corresponded to *Aspergillus fumigatus*; **C** and **D** corresponded to *Rhizopus arrhizus*. Hyphae can artefactually seem to have septae because tissue can fold over itself during processing, which can create artificial lines that can be confused with septations. Similarly, the historically described 90° branching angle of Mucorales in tissue, versus 45° branching angle of septate moulds, can be difficult to identify in tissue due to interstitial pressures exerted on the fungi by the tissue and alterations in tissue architecture during processing. Thus the wider and irregular (ribbon-like) nature of the hyphae are more reliable distinguishing characteristics than septations and angle of branching [4]

Various diagnostic methods extant constitute of biopsy for microbiological culture, conventional microscopic examination, specific histopathologic staining, antigen–antibody reaction, molecular testing, and antifungal susceptibility assay (Additional file 1: Table S1). Notwithstanding, not all fungi can be recognized via any one given method; and proper diagnostic aids depend on the epidemiology of prevalent fungal infection in the locality as well as the available laboratory tests. In ambiguous cases, fungal culture, microscopy of hematoxylin and eosin staining, and polymerase chain reaction test are often useful adjuncts highlighting the role of multiple diagnostic methods [74]. The diagnosis of oromaxillofacial invasive mucormycosis though sometimes difficult can be simplified by a team approach having specialists of different fields. For our cases, the conclusive diagnosis of invasive mucormycosis is ultimately received through histopathological analysis after taking tissue biopsy that identifies extensive tissue necrosis and the presence of broad (5–20 μm or more in width), thinned-wall fungal hyphae, which irregularly branched and occasionally have a ribbon-like appearance. These numerous nonseptate hyphae invaded the lumen of a large blood vessel, causing thrombosis, typical for invasive mucormycosis (Fig. 7). In addition, high power photomicrograph of the fungal hyphae stained by fluorescence can be more depictive of the characteristic microscopic appearance (Fig. 8).

The clinical progression of mucormycosis usually precedes its radiological appearances. Displaying on non-contrast CT scans of coronal with axial reformats has the best visualization [75, 76]. Maxillofacial mucormycosis commonly shows with pansinusitis or multiple sinuses involvement, which is oftentimes unilateral but can be quickly aggressive to bilateral components. Its CT images present with air-fluid levels, enlargement in mucosal thickness, and bone erosion; however that is s not sufficiently precise to help distinguish mucormycosis from maxillary malignancies [77].

**Fig. 9:**
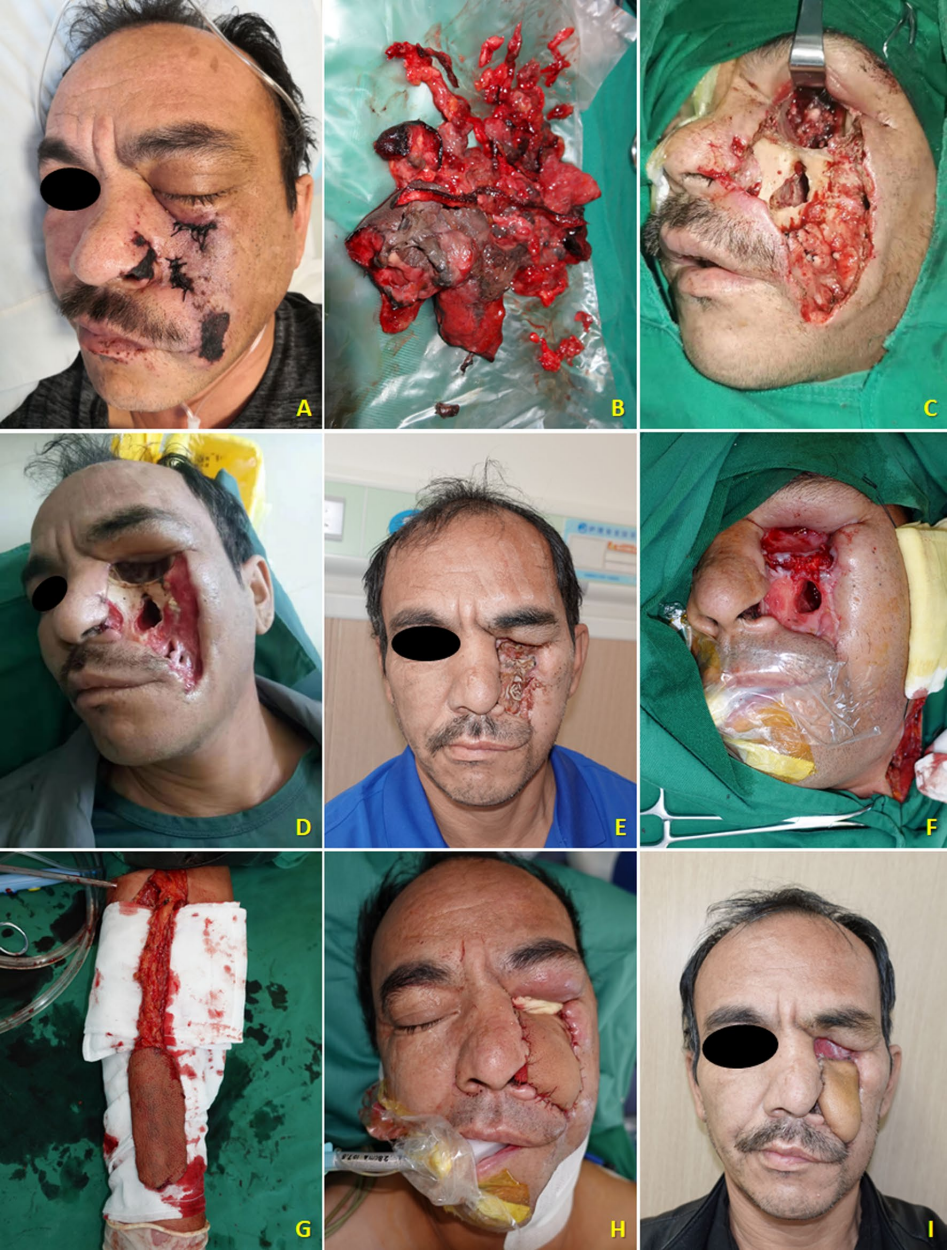
Typical case in our patients suffering from maxillofacial mucormycosis. Due to the disease’s high affinity to the arteries and its internal elastic lamina causing embolism and infarctions. Previous imaging results revealed opacification of the left maxillary sinus, ethmoid sinus, and frontal sinus, erosion of the anterolateral wall of left maxillary antrum with thickening of the sinus lining, embolus formation in left facial artery and ophthalmic artery. **A** Preoperative physical sign showed orbital and facial cellulitis with areas of necrotic skin; **B** Massive necrotic infected tissue removed; **C** The first surgical debridement; **D** Clinical aspect 2 weeks after operation; **E** Follow-up photo documentation (wound covered by Vaseline gauze) after 5 months; **F** The second surgical debridement along with reconstruction for disfiguring maxillofacial deformity; **G** The pedicled forearm flap was harvested; **H.** The skin island reconstructed the defect cavity; **I** Postoperative view in the follow-up of 45 days

Contemporary treatment strategy for mucormycosis is principally surgical management, which should be performed whenever feasible in parallel to antifungal drugs. Various authors have reported higher cure and survival rates through surgical interventions [73, 78–82]. Successful regime counts on timing, but it also should be noted that many patients may be too sick to undergo surgery. Oromaxillofacial invasive mucormycosis should be treated by radical surgical debridement with margins clear of infection, although it is currently unclear how to define such margins. Identifying margins of infected borders during the surgical procedure may be achieved in real time using fluorescent brightener on the resected tissue [41]. This approach limits unnecessary resection of non-infected tissue in craniofacial areas. Complete debridement, including endoscopic debridement or excision of infected tissues, increased survival rates in a cohort of solid organ transplant recipients [83]. It is imperative to perform a biopsy of affecting area and initiate intravenous antifungal treatment, once the clinical suspicion has increased [84]. Surgical operation should be conducted with a sense of urgency so as to limit the fulminant spread of infection to contiguous structures. Adapting the extent of surgery to the distribution of mucormycosis improves outcome and reduces unnecessary loss of healthy tissue [82]. Thorough cut out of the infected sinuses as well as sufficient debridement of the retro-orbital space (fatty tissue) can effectively prevent necrotic infection from disseminating into the eye, thereby improving the cure rate. Currently, debridement extends until clean tissue is seen, but no intraoperative microscopic evaluation is done. Patients need to be closely followed after surgery to identify new necrosis, which must be managed by repeated debridement. Radical resection or repeated debridement of lesion is regularly recommended with subsequent reconstructive surgery [85, 86] (Fig. 9). Few scientific literatures proposed treatment about the surgical technique of reconstruction for this disease. There are a multitude of considerations and perioperative measures that aim to maximize the success of tissue transfer, including acknowledgment of psychological and psychiatric factors, tight glycemic control, nutritional support, intraoperative surgical technique, and close postoperative monitoring of the patients' hemodynamic physiology [87]. The defect resulted from radical surgical debridement is extensive, requiring reconstructive surgery with a pedicled than free flap. Additionally, given that the fungal affinity to blood vessels as well as poor blood circulation of free flaps, were not considered for reconstruction due to be short of a continuous arterial inflow and venous outflow [50]. Thus, a pedicled forearm flap was designed and performed [88] (Fig. 9). This harvested flap was elevated and turned over the disfigurement site. The skin island filled the defect cavity, and a skin graft covered the muscle deep surface to restore maxillofacial continuity (Fig. 9).

Early initiation of antimycotic intervention at the very first likelihood of acute invasive maxillofacial mycotic pathology can reduce mortality rate to a certain extent [89]. Amphoterecin B (AmB), either conventional or liposomal, is probably the mainstay of treatment that has been successfully applied in zygomycosis [90]. Intravenous high-dose (1.0–1.5 mg/kg/day) AmB deoxycholate was widely administrated; however, lately liposomal AmB (5–10 mg/kg, daily) with lower nephrotoxicity has become the empirical drug of choice in *Mucor* species management [91, 92]. Even so, some trials have raised concerns over its evidence of dose-dependent hepatotoxicity [93]. Moreover, high drug costs of liposomal AmB also remains the biggest obstacle in its prolonged use, despite it proves stable activity against Mucorales. Apart from that, medical management alone is not effective because of poor drug delivery to the infection site due to extensive vascular thrombosis [94]. Posaconazole is the secondary drug of choice (recommended dosage: 800 mg/day in 4 divided doses) in treating mucormycosis as a promising newer azole approved by the US FDA (Food and Drug Administration) [95]. A few articles reviewed have taken posaconazole as a combined medicine for curing maxillofacial invasive mucormycosis [18, 20, 23, 39, 42]. Considering it can be taken orally with low incidence of side effects and is hence excellent for prolonged outpatient regimes. First-line treatment with high-dose liposomal AmB is strongly recommended, while intravenous isavuconazole and intravenous or delayed release tablet posaconazole are recommended with moderate strength. Both triazoles are strongly recommended salvage treatments. AmB deoxycholate is recommended against, because of substantial toxicity, but may be the only option in resource limited settings [4].

Use of adjuvant hyperbaric oxygen therapy has a direct fungicidal effect and has been reported as an effective adjunct in some comprehensive regimes especially in patients with diabetic ketoacidosis-induced mucormycoses [96, 97].

## Conclusion

In conclusion, the uncommon phenomenon of cranio-maxillo-facial invasive mucormycosis may be associated with fatal disease. Better recognition this condition, by avoiding both unnecessary investigations and possible misdiagnosis could result in more timely treatment. Mucormycosis should be known to the dental practitioner and maxillofacial surgeon, especially in any underlying predisposing disorder (e.g. diabetic individuals and other immunosuppressed patients). For this life-threatening infection that is considered to be a medical emergency, prompt and aggressive intervention with a combination of ablative surgery, intravenous antifungal treatment as well as hyperbaric oxygen therapy, along with simultaneous appropriate elimination of underlying causes of immunosuppression and risk factors should be recommended.

## Supplementary Information

**Additional file 1: Table S1.** Overview of laboratory methods available for the diagnosis of oromaxillofacial invasive mucormycosis.

**Additional file 2: Fig. S1.** Risk factors for invasive oromaxillofacial mucormycosis.

## Data Availability

The analyzed data sets generated during the study are available from the corresponding author on reasonable request.
