# Supplementary material for: A retrospective analysis for the management of oromaxillofacial invasive mucormycosis and systematic literature review
Source: BMC Oral Health. 2023 Feb 21;23:115. doi: 10.1186/s12903-023-02823-4 (PMC9942087; doi:10.1186/s12903-023-02823-4)
Supplement: Supplementary file 1 — Additional file 1: Table S1. Overview of laboratory methods available for the diagnosis of oromaxillofacial invasive mucormycosis. [file 12903_2023_2823_MOESM1_ESM.docx]

Supplementary Table 1. Overview of laboratory methods available for the diagnosis of oromaxillofacial invasive mucormycosis.

| **Category** | **Methodological description** |
| --- | --- |
| Direct microscopy (H&E*) | While H&E-stained sections are usually adequate to identify fungal elements, use of special stains makes it easier. It is determined whether the disease is invasive or noninvasive based on evidence of invasion of fungal structures into the mucosa, associated vascular entities leading to thrombosis and tissue infarction and invasion into underlying bony structures. |
| Special histochemical stains (GMS**; PAS***) | Stainings like GMS and PAS make the fungal wall identifiable based on the presence of polysaccharides. These tests though sensitive are not specific and hence the fungal structures need to be carefully identified. GMS has been described to be the most sensitive stain and it has been recommended that a negative diagnosis of fungal pathology should not be given unless GMS staining is performed. |
| Serology  (1→3)-β-D-glucan panfungal test; Antigen and antibody based assays for non-European mycoses | Conventional microscopic and microbiological techniques remain the cornerstone of diagnosis but lack sensitivity. Cultures are often time-consuming as fungi are slow-growing and can yield false results. Since fungi are a part of the human microbiome, antigen and antibody detection-based serological assays are also not very useful as they commonly give false positive results. Antigen assays like galactomannan and glucan tests are used but are helpful only in specific pathogens. |
| Molecular methods  (FISH****test; PCR*****assays) | Molecular assays like FISH test and PCR hold a lot of promise but are very expensive and still need clinical validation. |

*H&E = Hematoxylin and eosin stain.

**GMS = Grocott’s-Gomori’s methenamine silver

***PAS = Periodic acid Schiff

****FISH = Fluorescence in situ hybridization

*****PCR = Polymerase chain reaction
