# Supplementary figures and images for: A retrospective analysis for the management of oromaxillofacial invasive mucormycosis and systematic literature review
Source: BMC Oral Health. 2023 Feb 21;23:115. doi: 10.1186/s12903-023-02823-4 (PMC9942087; doi:10.1186/s12903-023-02823-4)

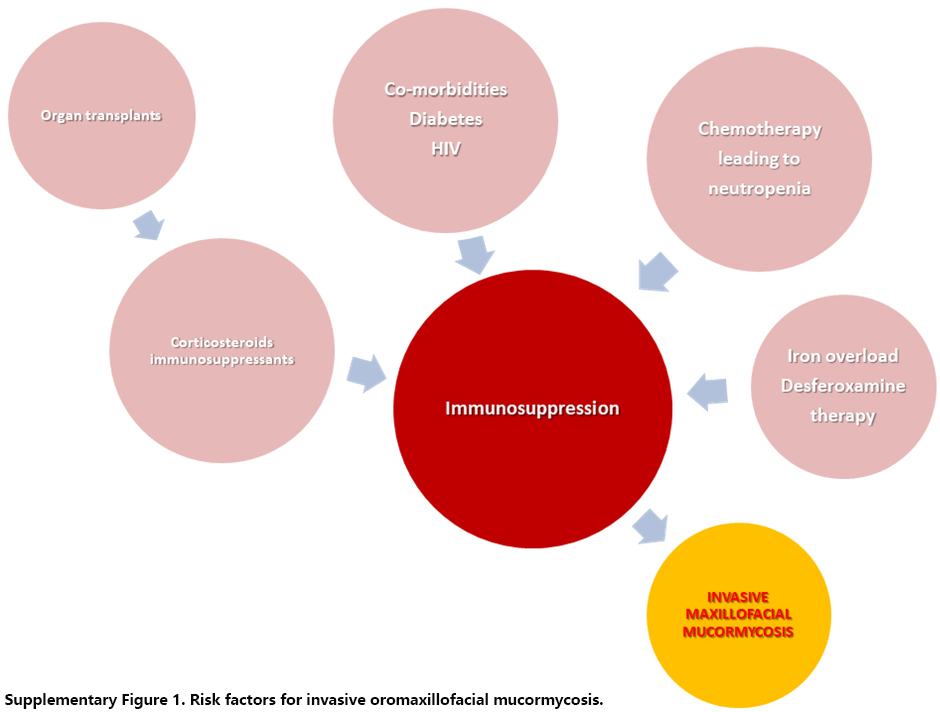

Supplement: Supplementary file 2 — Additional file 2: Fig. S1. Risk factors for invasive oromaxillofacial mucormycosis. [file 12903_2023_2823_MOESM2_ESM.tif]
